# Current attitudes and self-rated abilities of Bosnia and Herzegovina veterinarians toward pain recognition and quantification in domestic animals

**DOI:** 10.1017/awf.2023.46

**Published:** 2023-07-24

**Authors:** Nermina Spahija, Ismar Lutvikadić, Adna Ćoso, Alan Maksimović

**Affiliations:** University of Sarajevo Veterinary faculty, Zmaja od Bosne 90, 71000 Sarajevo, Bosnia and Herzegovina

**Keywords:** animal welfare, domestic animals, pain quantification, pain recognition, pain scales, questionnaire

## Abstract

In previous years interest has grown in investigating the attitudes and capabilities of veterinarians regarding the recognition, quantification and treatment of animal pain throughout different parts of the world and encompassing various species. This is the first report exploring the attitudes and self-rated abilities of veterinarians in Bosnia and Herzegovina (B&H) concerning recognition and quantification of pain in domestic animals. A study questionnaire was made available to 535 general practice veterinarians throughout B&H and 73 (14%) responded in full. The questionnaire contained polar, multiple choice, ordinal and interval scale questions and consisted of sections asking about demographic data, attitudes to pain recognition and quantification, use and availability of analgesics, estimates of pain intensity during specific surgical procedures, and the perceived need for pain assessment and continuing education programmes for analgesia. Half of the respondents considered the recognition and quantification of pain to be difficult while 89% did not make use of pain assessment scales. Of the respondents, (33/73; 45%) felt a certain level of pain to be advantageous since it reduces the activity of the healing animal, whereas 52% (38/73) did not agreed with this concept. Cost was a consideration when deciding whether or not to use analgesics for 58% (42/73) of the respondents with the most commonly used types being NSAIDs (72/73;99%) and opioids (60/73; 82%). Practitioners in B&H displayed awareness of the importance of pain assessment and management however a significant proportion were unaware of pain scales and relied upon physiological indicators of pain.

## Introduction

Pain is defined by the International Association for the Study of Pain (IASP) as “An unpleasant sensory and emotional experience associated with or resembling that associated with, actual or potential tissue damage” (Raja *et al.* 2020). The consequences of untreated pain are well documented in veterinary medicine (Tannenbaum 1999; Rutherford 2002). Pain leads to changes in animals’ physiology and behaviour, directly impacting their welfare and quality of life. It causes suffering and distress in animals, delays recovery from illness, injury, or surgery by interfering with the healing process, suppresses the immune system increasing susceptibility to infections. Pain influences the functionality of various different body systems through alterations in the secretion of hormones, neurotransmitters and enzymes pain (Jirkof 2017). Untreated or poorly managed pain can lead to maladaptive pain (Anderson & Muir 2005). In considering these negative aspects of pain, the American Pain Society (APS) started the ‘Pain as the fifth vital sign’ campaign in 1996 with the aim of increasing awareness in pain recognition, quantification and therapy (Levy *et al.* 2018). The endorsement of pain recognition and subsequent treatment has far reaching implications as regards the quality of care provided in human and veterinary hospitals (Mich *et al.* 2010). Pain assessment should therefore form a vital component of every animal’s physical examination such that a pain score may be considered the ‘fourth vital sign’ after body temperature, pulse rate, and respiratory rate (Epstein *et al.* 2015). Despite the clear emphasis on the importance of pain recognition, quantification, and management, the attitudes toward pain vary significantly within veterinary professionals and can be influenced by a variety of different factors, such as age and gender (Coleman & Slingsby 2007). In recent years, there has been growing interest in investigating the attitudes and capabilities of veterinarians towards the recognition, quantification and treatment of pain throughout different parts of the world and in a range of animal species. According to the literature, studies have been conducted in France, Finland, New Zeeland, Belgium, Brazil, Canada, Columbia, Germany, and Slovenia incorporating multiple animal species or specifically related to dogs and cats, horses, horses and cows, or only cows (Raekallio *et al.* 2003; Hugonnard *et al.* 2004; Williams *et al.* 2005; Dujardin & van Loon 2011; Lorena *et al.* 2013; Beswick *et al.* 2016; Morales-Vallecilla *et al.* 2019; Tschoner *et al.* 2020; Tomsič *et al.* 2021). Identification of the presence of pain and assessing its intensity remains a challenging task for veterinarians, presenting specific challenges in different animal species. Firstly, each species may exhibit unique pain responses and behavioural cues, making it difficult to establish consistent methods of assessment. Secondly, animals’ inability to verbally communicate their pain necessitates a reliance on indirect indicators, such as changes in behaviour, physiology, and facial expressions. Additionally, numerous species-specific anatomical and physiological differences exist which require the adaptation of pain assessment techniques to enable accurate evaluation across various animals. For cats the main issue with pain assessment is the difficulty of accurately interpreting and evaluating their subtle pain behaviours despite their tendency to conceal signs of discomfort (Monteiro *et al.* 2023). Horses, cattle and sheep (as prey animals) also tend to mask signs of pain and can be influenced by environmental and husbandry factors (Ashley *et al.* 2005; McLennan 2018). Determining pain in dogs is generally considered easier compared to other animal species due to their well-documented pain-related behaviours, vocalisations, and numerous studies providing validated pain assessment tools (Reid *et al.* 2018). Regardless of animal species, pain assessment is complicated by the involvement of an affective component as well as the sensory nervous component (Broom 2014). Pain is a complex phenomenon. Reliable, valid, and feasible measurements are challenging, especially considering the subjective nature of the response to pain (Broom 2014; McLennan *et al.* 2019). Furthermore, changes in behaviour are not always specific for pain in domestic animals (Anil *et al.* 2002). Notwithstanding challenges discussed in the literature regarding difficulties evaluating analgesic efficacy, further issues include differences in pain assessement for various animal species, species-specific drug metabolism, adverse effects, lack of approved drugs, financial constraints and client awareness about analgesia (Livingston 2010). The objective of this study was to investigate, for the first time, the attitudes of veterinarians in Bosnia and Herzegovina (B&H) regarding recognition, quantification, and management of pain in horses, cattle, sheep, dogs and cats (being the most common animals treated by veterinarians in this country) and to compare them with those reported in other countries. The need for continuing education programmes in pain recognition and analgesia in different animal species was also investigated through this survey. To our best knowledge there have been no publications regarding pain control and/or analgesic use in domestic animals in B&H.

## Materials and methods

### Questionnaire

#### Development and implementation

A request was sent via email to 535 general practice vets throughout B&H inviting them to participate in the study. The email contained a link to the study questionnaire and recipients’ addresses were obtained via the Veterinary Faculty University of Sarajevo records with a contact list representing all the veterinary practices in the country. All participants were veterinarians employed in clinics throughout B&H and the questionnaire was created in Bosnian (see Supplementary material) using the Google Forms platform written in form of polar, multiple choice, ordinal or interval scale questions. Respondents gave their consent for the use of their answers in this study, with the proviso that their identity would remain anonymous.

#### Questions

The questions were based on data from previously published studies (Capner *et al.* 1999; Lascelles *et al.* 1999; Raekallio *et al.* 2003; Hugonnard *et al.* 2004; Williams *et al.* 2005; Lorena *et al.* 2013; Beswick *et al.* 2016) and consisted of six sections. Section 1 contained data regarding sex (male, female), year of graduation (2001–2010, 2011–2020, etc), highest academic/specialisation qualification (masters, PhD), and type of practice (mixed animal practice, small animal practice, large animal practice). Questions on general pain assessment were contained in section 2. Veterinarians were asked about their ability to recognise and quantify pain, the methods they use for pain recognition and quantification, and the factors influencing their judgment (animal species and breed, individual sensitivity, previous pain experience). The ability to recognise and quantify pain was classified as excellent, sufficient, moderate, and insufficient. The use of behavioural and physiological parameters for pain recognition and familiarity with different pain scales (visual analogue pain scale, numerical rating pain scale, simple descriptive pain scale, composite pain scale, behavioural pain scale) and their use in everyday practice were examined. Section 3 was divided into subsections, and veterinarians were asked about pain assessment in horses, cattle, sheep, dogs, and cats. Veterinarian opinions about owners’ ability to assess pain in terms of their interpretation of behaviour and the reliability of their assessment were collected in section 4. Section 5 contained questions concerning pain management. Veterinarians were asked about specific analgesics and their use (NSAID and opioids listed in Supplementary material along with all other sections described), local anaesthetics (lidocaine, bupivacaine, mepivacaine), local anaesthetic techniques (local and epidural anaesthesia), and alternative methods for pain relief (acupuncture, homeopathy, physical therapy). Factors influencing the choice and use of analgesics were examined (price, available information, potential side-effects, analgesic potency, market availability). Analgesic protocols for most common surgical procedures (ovariohysterectomy and orchiectomy) were evaluated, with usage categorised as always, often, sometimes or never, and single or multiple applications. Judgment about pain severity for these surgical procedures was obtained, predefined as mild, moderate, or intensive. In section 6, veterinarians ranked their knowledge of pain management on a scale from 1 to 5 (1: poor; 2: weak; 3: good; 4: very good; 5: excellent). The primary source of information regarding pain assessment and treatment was also sought with five potential sources listed: veterinary schools, journal articles, personal experience, regional and national meetings, and online webinars. At the end of this section, veterinarians were invited to provide any further comments, opinions, or recommendations on the subject.

### Statistical analysis

The study data were collected in a spreadsheet and analysed using Microsoft Office Excel®. Descriptive statistics were calculated. For nominal variables, frequencies and percentages were used.

### Ethical approval

This work was approved by the ethics committee of the Veterinary Faculty University of Sarajevo under the reference number 01-02-18-5/20.

## Results

A questionnaire link was sent to a total of 535 veterinarians with a response rate of 14% (73 questionnaires). Four of the questionnaires were returned incomplete (1 of 4) or blank (3 of 4). The incomplete questionnaire contained only two answers, pertaining to the respondent’s gender and year of graduation. Blank answers present in other questionnaires were solely in relation to the type of animal(s) that the veterinarian had no dealings with. Data collection lasted from November 2020 until April 2021. Study participants were predominantly male, accounting for 70% of the respondents and in terms of veterinary practice, the majority (68%) worked in mixed practice (Table 1).

**Table 1.** Demographic data of veterinarians in Bosnia and Herzegovina responding to the survey questionnaire on pain recognition and control

Characteristic	Distribution number (%)
Gender (73 responses)	
Male	51 (70)
Female	22 (30)
Year of graduation	
1980–2000	5 (7)
2001–2010	34 (47)
2010–2020	34 (47)
Postgraduate degree	
Masters	9 (12)
PhD	3 (4)
Type of practice	
Mixed animal practice	50 (68)
Small animal practice	20 (27)
Large animal practice	3 (4)

### Pain assessment

**Figure 1.**
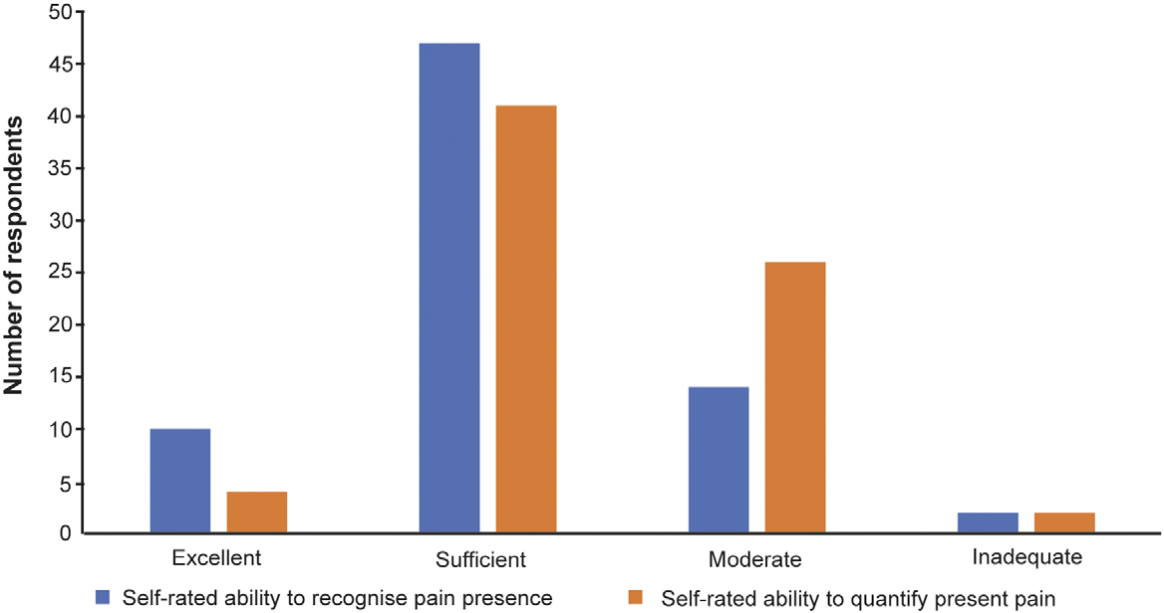
Self-rated ability to recognise and quantify the presence of pain by veterinarians in Bosnia and Herzegovina responding to the survey (n = 73).

Approximately half of the study respondents (37/73: 51%), considered it difficult to recognise and quantify pain and respondents’ self-rated abilities for pain recognition and quantification are shown in Figure 1. A significant proportion (65/73; 89%) do not use pain assessment scales. However, almost all participants (71/73; 97%), noted knowledge of normal animal behaviour facilitates pain assessment. Answers pertaining to different factors affecting animals’ pain experience are shown in Table 2.

**Table 2.** Factors with an effect on the pain experience of animal according to veterinarians in Bosnia and Herzegovina responding to the survey questionnaire (n = 73)

Factor	Number of responses (%)
Individual sensitivity	65 (89)
Previous painful experience	36 (49)
Animal species	31 (42)
Animal breed	25 (34)

Recognition and quantification of pain based on evaluation of both behavioural and physiological parameters are reported by 89% (65/73) of participants, behavioural parameters alone by 10% (7/73), and only physiological parameters by one respondent. The most useful behavioural and physiological pain indicators scored by the respondents using previously recommended pain scales for various animal species are shown in Tables 3 and 4. Sixty-six percent of respondents (48/73) felt assessment and interpretation of behavioural changes caused by pain could not be considered reliable if performed by the owner. Similarly, more than half of the respondents (46/73; 63%), considered adequate pain evaluation only able to be performed by veterinarians.

**Table 3.** Behavioural pain indicators considered useful for various animal species by veterinarians in Bosnia and Herzegovina responding to the survey questionnaire (n = 73)

Behavioural pain indicators	In horses (n = 60)	In cattle (n = 62)	In sheep (n = 50)	In dogs (n = 73)	In cats (n = 73)
Demeanour	47 (78%)				
Mental status				49 (67%)	39 (53%)
Behaviour of the dog on a leash				51 (70%)	
Position in the room				49 (67%)	
Appetite	39 (65%)	45 (73%)		47 (64%)	45 (62%)
Comfort					30 (41%)
Locomotion		50 (81%)			
Activity		45 (73%)		48 (66%)	39 (53%)
Pawing	45 (75%)				
Kicking/stepping with their feet		44 (71%)			
Kicking in the abdomen	50 (83%)				
Rolling	44 (73%)				
Sweating	32 (53%)				
Stretching	29 (48%)	18 (29%)			
Vocalisation	20 (33%)			40 (55%)	56 (77%)
Posture (weight distribution)	21 (35%)				42 (57%)
Lateral recumbence	10 (17%)				
Head below spine		32 (52%)			
Back curvature during rest		30 (48%)			
Head and neck extension while laying down		28 (45%)			
Head on the ground while laying down		27 (44%)			
Partial or complete extension of hind limbs during ventral recumbence		27 (44%)			
Interactive behaviour (aggression towards foal and other horses)	20 (33%)				
Interactive behaviour (aggression towards handlers)	18 (30%)				
Interactive behaviour (reaction to observer[s], sound stimulus)	11 (18%)	24 (39%)			
Interactive behaviour (interaction with other animals)		12 (19%)			
Tail flicking (excluding flicking to chase off insects)	15 (25%)	16 (26%)			
Licking of surgical wounds		14 (23%)			
Reaction to wound/painful area palpation	46 (77%)			62 (85%)	61 (85%)
Reaction to abdomen palpation					56 (77%)
Facial expressions	35 (58%)				34 (47%)
Spasm of masticatory muscles			30 (60%)		
Abnormal ear position			29 (58%)		
Abnormal lip and jaw profile			22 (44%)		
Orbital muscles tension			21 (42%)		
Abnormal shape of the nostrils and nasal philtrum			20 (40%)		
Tears	16 (27%)				
Self-mutilation					44 (60%)
Collapse	27 (45%)				
Stupor	6 (10%)

**Table 4.** Physiological parameters as pain indicators in various types of animals considered useful by veterinarians in Bosnia and Herzegovina responding to the survey questionnaire (n = 73)

Physiological parameters as pain indicators	In horses (n = 59)	In cattle (n = 57)	In dogs (n = 67)	In cats (n = 64)
Heart rate	38 (64%)	45 (79%)	55 (82%)	53 (83%)
Respiratory rate	37 (63%)	47 (82%)	58 (87%)	56 (87%)
Rectal temperature	17 (29%)	22 (39%)		
Blood pressure	4 (7%)		6 (9%)	5 (8%)
Digestive sounds	20 (34%)	31 (54%)		
Clinical exam	32 (54%)			
Type of condition	24 (41%)			
Dilated pupils			32 (48%)	
Salivation			30 (45%)	

### Pain management

All respondents consider analgesics beneficial for animals in pain, and (72/73; 99%) associate it with improved recovery. Nevertheless, 45% (33/73) believe a certain degree of pain to be advantageous because it reduces the activity of animals after trauma or surgery, while 52% (38/73) disagree with this statement. Only 3% (2/73) of respondents did not answer the question. The main issues concerning analgesic use were potential adverse effects (44/73; 60%) and cost (42/73; 57%). Factors identified as influencing the choice of analgesics were: market availability (65/73; 89%), analgesic potency (38/73; 52%), price (33/73; 45%), potential side-effects (27/73; 37%), and locally available information (23/73; 31%).

Owners specifically requiring analgesics for their animals is reported by 79% (58/73) of respondents. Drugs used for pain management by respondents in this study are presented in Table 5.

**Table 5.** Proportion of analgesic drug used to treat animals by veterinarians in Bosnia and Herzegovina responding to the survey questionnaire (n = 73) (multiple choice)

Drugs (overall)	N (%) Result into the group (%)
**Opioids**	**60 (82)**
Tramadol	57 (95)
Butorphanol	8 (13)
Methadone	6 (10)
Buprenorphine	4 (7)
Pethidine and morphine	0
**NSAIDs**	**72 (99)**
Carprofen	64 (89)
Ketoprofen	41 (57)
Flunixin	33 (46)
Phenylbutazone	21 (29)
Meclofenamic acid	3 (4)
Dipyrone/hyoscine and vedaprofen	2 (3)
Eltenac and toclofenamic acid	0
**Local anaesthetics**	**70 (96)**
Lidocaine	70 (100)
Mepivacaine	3 (4)
Bupivacaine	2 (3)
**Other**	
Xylazine	61 (84)
Ketamine	58 (79)
Detomidine	10 (14)
Romifidine	2 (3)
Others (respondents did not specify other used sedatives)	13 (18)

Epidural administration of local anaesthetics is reported by 41% (30/73) of respondents; 93% (28/30) for use on cattle and 7% (2/30) in dogs. Lidocaine and xylazine for epidural anaesthesia/analgesia were used by 83% (25/30) and 23% (7/30) respondents, respectively. Morphine, tramadol, bupivacaine, mepivacaine, or detomidine were not used for this purpose. Indications for epidurals were standing surgery (24/30; 80%), uterine prolapse (20/30; 67%), perineal and rectal laceration (15/30; 50%), perineal surgery (11/30; 37%), hind-limb injuries (8/30; 27%), foaling, dystocia, and rectovaginal fistula (3/30; 10%).

Use of other methods of pain relief are reported by 37% (27/73) of total evaluated surveys. Here, physical therapy was described in 44% (12/27), homeopathy in 11% (3/27), acupuncture in 4% (1/27), and other methods in 59% (16/27) of surveys.

**Figure 2.**
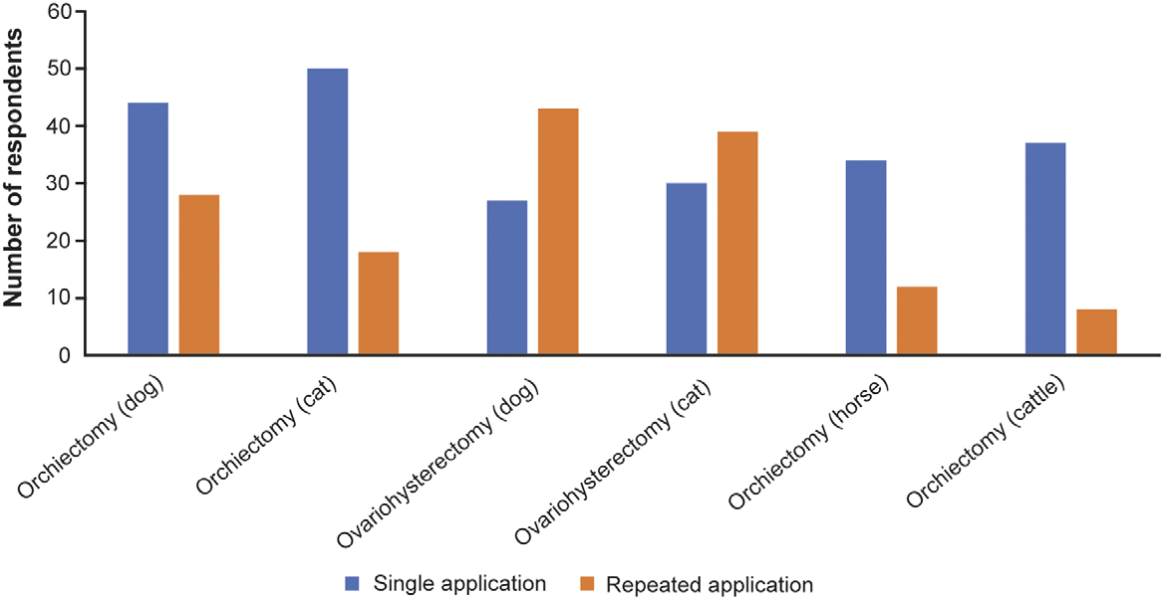
Analgesia practiced by veterinarians in Bosnia and Herzegovina responding to the survey (n = 73) for animals undergoing ovariohysterectomy or orchiectomy.

**Figure 3.**
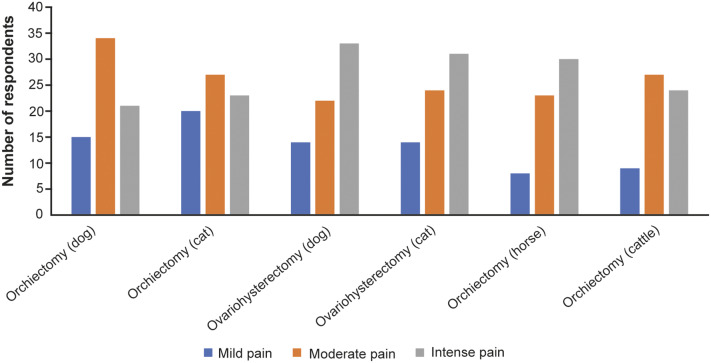
Assessment of pain intensity for ovariohysterectomy and orchiectomy by veterinarians in Bosnia and Herzegovina responding to the survey (n = 73).

Analgesia for animals undergoing ovariohysterectomy or orchiectomy and judgment of pain intensity for these procedures, are presented in Figures 2 and 3, respectively.

Respondents indicated what they considered their level of knowledge on the subject of pain treatment on a scale from 1 to 5. Accordingly, (4/73; 5%) considered it to be 5, (22/73; 30%) as 4, (41/73; 56%) as 3, (5/73; 7%) as 2, and only (1/73; 1%) as 1. Most of the respondents considered their knowledge of pain treatment to be gained from personal experience in practice (65/73; 89%) and academic studies (61/73; 83%), followed by professional journals (34/73; 47%), regional and national meetings (22/73; 30%), and online webinars (15/73; 20%).

## Discussion

This is the first study reporting veterinarians’ attitudes in B&H toward pain recognition and quantification in domestic animals. Most of the respondents stated the knowledge they acquired during undergraduate studies as essential in pain assessment and management, followed by professional journals, regional and national meetings, and online webinars. In contrast, many other similar studies reported conferences and seminars as being the primary source of knowledge, followed by scientific and professional articles, and communication with colleagues, while university studies were among the least used sources of information on pain management (Williams *et al.* 2005; Lorena *et al.* 2013; Beswick *et al.* 2016; Morales-Vallecilla *et al.* 2019; Tomsič *et al.* 2021).

The low response rate mirrors investigations by Tomsič *et al.* (2021), Williams *et al.* (2005) and Price *et al.* (2002). In the present study, the low response rate could be as a result of inadequate awareness regarding the importance of pain recognition and quantification for improving quality of care and pain management in animals. Nevertheless, the amount of time required to complete the questionnaire almost certainly contributes to the low turn-out of participants. We can assume a greater representation of veterinarians who consider this a worthwhile topic since time would need to have been set aside from daily practice or private obligations to complete the questionnaire. Although this online survey represents the opinion of only a small percent of B&H veterinarians, the demographic of the survey participants showed consistency with the current overall profile of the B&H veterinary profession, thereby suggesting the results to be on the whole representative of the veterinary profession in B&H.

### Pain assessment

In our study, the majority of veterinarians considered their ability in pain recognition and quantification as sufficient. These results are similar to studies conducted in New Zealand (Williams *et al.* 2005), Ontario (Beswick *et al.* 2016), and Colombia (Morales-Vallecilla *et al.* 2019). Additionally, most veterinarians in France considered their knowledge sufficient in pain recognition but inadequate in pain quantification (Hugonnard *et al.* 2004). Contrary to the above results, most veterinarians from the Netherlands considered their knowledge insufficient or moderate (Lascelles *et al.* 1999; Dujardin & van Loon 2011; Lorena *et al.* 2013). Swiss veterinarians expressed weakness when evaluating pain severity (Perret-Gentil *et al.* 2014) while 42% of the respondents in a Queensland study reported difficulties recognising pain in dogs (Weber *et al.* 2012). It is known that pain assessment can depend on the observer’s experience (Tschoner *et al.* 2020). In our study, respondents were almost equally divided regarding year of graduation (Table 1). Therefore, variability between observers should also be considered in pain assessment methods (Mathews 2000). Interestingly, there is some evidence that medical practitioners with personal experience of intense pain display greater empathy towards patients in a painful state (Holm *et al.* 1989).

Our study showed similar results as work carried out in France which found that most veterinarians ignored the existence of pain scales (Hugonnard *et al.* 2004). One-fifth of Queensland veterinarians used formal pain scales (Weber *et al.* 2012). Veterinarians in Colombia who participated in continuing education programmes were more likely to use pain scales and were confident when assessing pain (Morales-Vallecilla *et al.* 2019). Pain assessment scales improve the ability of observers to recognise and quantify pain in animals, as well as the assessments made by different observers. A continuous evaluation of patients based on results obtained using a pain assessment scale increases the awareness of pain (Wagner 2010). In human medicine, increased awareness of pain and an increase in the administration of analgesics were noted when a printed pain assessment form was provided to nurses (Mathews 2000). There is also the potential confusion created by a language barrier: simple literature translation of a previously validated pain scale in one language does not make it valid in another language and culture, resulting in a lack their use (Della Rocca *et al.* 2018).

Veterinarians in this study agreed that an awareness of the animal’s usual behaviour facilitates pain assessment based on a combination of behavioural and physiological parameters. A small number of veterinarians used only physiological parameters for pain assessment even though physiological parameters are not considered reliable as pain indicators (Mathews 2000). Studies have not found any correlation between pain presence and intensity and heart rate (Price *et al.* 2003; Graubner *et al.* 2011). Excluding specific signs of abdominal distress or traumatic injury, respondents nominated demeanour as being the most useful behavioural indicator of pain and heart rate the most useful physiological indicator which is comparable to a UK study (Price *et al.* 2002). Evaluation of pain in cattle utilises behavioural changes (Molony *et al.* 1995; Gleerup *et al.* 2015; Tschoner *et al.* 2020), and in this study, the most common of those assessed were locomotion, activity, appetite, and kicking/stepping with their feet. Changes in facial expression caused by pain have proven a reliable, accurate, and valuable assessment tool in humans and animals (McLennan *et al.* 2016; Evangelista *et al.* 2019). This is related to the results of our study where respondents assessed pain in sheep using masticator muscle spasms and abnormal ear position as leading indicators of pain. Specific changes in facial expression for pain assessment in sheep have been recognised and described by McLennan *et al.* (2016) in which they developed a facial expression scale using foot-rot and mastitis as models of pain. Almost half of the respondents made use of facial expressions for pain assessment in cats, similar as for pain assessment in horses. Comparable to other studies (Hugonnard *et al.* 2004; Weber *et al.* 2012; Beswick *et al.* 2016; Morales-Vallecilla *et al.* 2019; Tomsič *et al.* 2021), reaction to surgical wound palpation was considered one of the most valuable indicators of pain in dogs and cats, followed by depression and abnormal posture. Vocalisation is considered as an important indicator of pain by veterinarians in this study, comparable to a survey conducted in Ontario. According to Beswick *et al.* (2016), vocalisation was the second most commonly used indicator of pain after demeanour, followed by heart rate, but less sensitive than heart rate and response to palpation. French veterinarians did not consider vocalisation as a significant indicator of acute pain, and the reason for this may be because they associate it more with stress or distress (Hugonnard *et al.* 2004). Vocalisation was assigned higher scores in cats than in dogs as a pain indicator in our and other studies (Hugonnard *et al.* 2004; Weber *et al.* 2012; Beswick *et al.* 2016; Morales-Vallecilla *et al.* 2019).

The majority of veterinarians in the present study agreed that the owners cannot reliably assess and interpret pain behaviour. A similar opinion was expressed in Finnish veterinarians, even if they considered that owners or animal handlers may recognise behavioural changes that would otherwise go unnoticed without necessarily seeing them as pain indicators (Raekallio *et al.* 2003). However, in dogs and cats it is considered that behavioural changes associated with chronic pain may be detected only by someone very familiar with the animal, and that is usually the owner. In cats, especially, owner assessment is the mainstay of the assessment of chronic pain (Mathews *et al.* 2014).

### Pain management

All our respondents considered that animals benefited from pain relief, and almost all that recovery is better if the animal has received analgesics. Still, nearly half felt a certain degree of pain to be beneficial since it reduces movement post-surgery. Similar results were found for studies conducted in the UK, New Zealand, and Finland (Capner *et al.* 1999; Raekallio *et al.* 2003; Williams *et al.* 2005). Short (1998) noted a longer recovery time, reduced intake of water and food with signs of distress if surgical procedures were performed without anaesthesia or adequate analgesia and anaesthesia. According to the American College of Veterinary Anaesthesiologists, there are no beneficial effects of pain in veterinary medicine (Raekallio *et al.* 2003). In our study, most of the respondents scored ovariohysterectomy of dogs and cats as procedure of intensive pain. Yet many respondents used single dose analgesic application as pain management for this surgical procedure in dogs and cats. The results indicate that dogs are more likely to get analgesics multiple times compared to cats. In contrast, orchiectomy is estimated by study respondents as moderately painful procedure in all animal species with the exception of horses for which the pain was considered intense. A single dose of analgesic is observed as a general trend for orchiectomy pain management in all animal species by our respondents.

The majority of our respondents agreed that analgesic side-effects limit their use, while a study in Brazil showed that respondents disagreed with the statement that analgesic side-effects outweigh the benefits (Lorena *et al.* 2013). Analgesic use by veterinarians is related to the concern for potential or adverse reactions associated with its administration (Mathews 2000). A study conducted in Bavaria reported that despite a majority of veterinarians stating they use analgesics adequately, pain management was still low, especially for castration and dehorning in calves (Tschoner *et al.* 2020). The use of analgesics can be restricted on the grounds of availability, expense, or regulatory legislation guidelines (Morales-Vallecilla *et al.* 2019). Analgesic choice here was governed mainly by market availability. Respondents reported that owners requested analgesics, but that price impacted on choice. Almost all respondents agreed that analgesia was necessary with general anaesthesia and/or sedation. The low rate of local anaesthetic use, epidural techniques, and drugs shows the need for further education in local and regional anaesthesia techniques.

Similar to other studies, the most frequently used NSAIDs amongst veterinarians in B&H is carprofen, followed by ketoprofen, flunixin meglumine, and phenylbutazone. Tramadol was the most commonly used opioid. Most of the respondents in this study do not use epidural anaesthesia, and when used, it is used almost exclusively in large practice (cattle). Lidocaine and xylazine were the most common medications used for epidural anaesthesia. Mirroring these results, veterinarians in New Zealand do not tend to use epidural anaesthesia in a small animal practice (Williams *et al.* 2005). The use of other pain-relieving methods, such as physical therapy, acupuncture, and homeopathy, is yet to become established in B&H. The use of physical therapy is comparable with that found with Finnish veterinarians (Raekallio *et al.* 2003), use of homeopathy resembles that of Brazilian veterinarians (Lorena *et al.* 2013), while acupuncture use falls significantly short compared to these other countries studied.

### Animal welfare implications

Observations in this study urge the need for more effective access to information through open discussion and literature (preferable in the native language) and for continuing education programmes in recognition, quantification, and management of pain in animals for improving the quality of care. Successful translation of the pain scales into the native language, their evaluation and validation for specific animal species, would contribute significantly to their usage. In this way, a more intensive utilisation of adequate pain assessment methods and pain management would be achieved. Furthermore, the concerning finding here that not all veterinarians apply analgesia during ovariohysterectomy surgery, or the belief for some that a degree of pain is beneficial after surgery since it reduces movement serves as an indication that change is required. We recommend implementation of a national programme to aid familiarisation with pain in domestic animals; its deteriorative effects, adequate assessment methods and management itself.

## Conclusion

Practitioners in B&H showed awareness of the importance of recognition, quantification and management of pain in different animal species. Furthermore, they demonstrated high levels of interest in improving their current knowledge and skills. However, most (65; 89%) were unaware of pain scales, despite 64% (47) considered themselves to possess good pain recognition skills. The majority of our respondents ranked physiological parameters as the most valuable indicators of pain, regardless of animal species, even though physiological parameters are not considered to be reliable pain indicators. The realisation that not all veterinarians routinely use analgesics for surgical procedures, such as ovariohysterectomy, is a cause for concern.

## Supplementary material

For supplementary material accompanying this paper visit https://doi.org/10.1017/awf.2023.46. click here to view supplementary material
