# Supplementary material for: Current attitudes and self-rated abilities of Bosnia and Herzegovina veterinarians toward pain recognition and quantification in domestic animals
Source: Anim Welf. 2023 Jul 24;32:e50. doi: 10.1017/awf.2023.46 (PMC10936280; doi:10.1017/awf.2023.46)
Supplement: Supplementary file 1 [file S0962728623000465sup001.pdf]

QUESTIONNAIRE

Section 1 – Respondents' demographic data

1. Sex: Male Female
2. Year of graduation from the Faculty of Veterinary Medicine: …. – 2000; 2001 – 2010; 2011- 2020.
3. Highest academic title or title (year):
4. Type of practice: small animal practice, large animal practice, mixed animal practice.

Section 2 – General pain assessment

5. Do you find it difficult to assess pain in animals? YES NO
6. Your ability to detect the presence of pain is: very good; good; moderate; weak.
7. Your ability to quantify pain: very good, good, moderate, weak.
8. Do you use pain assessment scales: YES NO
9. To assess the presence of pain and its quantification pain you use (you can choose more than one answer): visual analogue table, numerical pain assessment table, simple descriptive table, composite pain assessment tables, behavioral pain assessment tables, I do not use pain assessment tables.
10. Does knowing the animal typical behavior facilitates pain assessment? YES NO
11. Do you think that the perception of pain is influenced by (you can choose more than one answer): type of animal, breed of animal, individual sensitivity, previous experience of pain and environment.
12. Do you assess the presence of pain and quantifiy it on the basis of: changes in behavior, changes in physiological parameters, a combination of the above.

Section 3 – pain assessment in horses, cattle, sheep, dogs and cats

Section 3.1. – Pain assessment in horses

13. When assessing pain in horses, if you assess pain based on behavior, mark the behaviors you pay attention to (it is possible to choose more than one answer):
- appearance (refusal to move, restlessness, anxiety, excitement, depression)
- facial expressions (position of ears, appearance of eyes and surrounding musculature, tension of chewing muscles)
- sweating
- kicking in the abdomen
- pawing
- tail wagging (do not include tail wagging to repel insects)
- position (carrying body weight, comfort)
- head movement
- appetite
- interactive behavior – pays attention to people
- interactive behavior – aggression towards the foal and other horses
- interactive behavior – aggression towards people
- response to palpation of the painful area

- presence of appetite
- rolling
- stretching
- vocalization
- teeth grinding
- tearing
- lateral position
- sopor (deep sleep)
- recumbency

14. When assessing pain in horses, if you assess pain based on physiological parameters, mark which ones (it is possible to choose more than one answer):
- heart rate
- respiratory frequency
- blood pressure
- digestive sounds
- rectal temperature
- the outcome of the clinical examination
- type of disorder

Section 3.2. – Pain assessment in cattle

15. When assessing pain in cattle, if you assess pain based on behavior, mark which behaviors you pay attention to (it is possible to choose more than one answer):
- interactive behavior – response to tactile, sound and/or visual stimuli
- interactive behavior – interaction with other animals nearby
- locomotion (way of moving, refusal to move or stop)
- activity (normal or reduced movement, restlessness, frequent changes from lying down to standing position)
- appetite
- sudden and constant wagging of the tail
- licking the surgical wound
- curvature of the back during rest
- kicking/stepping
- caudal stretching of the hind limbs during rest
- head below the line of the spinal column
- lying in a ventral position with full or partial extension of one or both hind limbs
- lying with the head on the ground
- neck and body extension while lying in the ventral position

16. When assessing pain in cattle, if you are assessing pain based on physiological parameters, mark which ones (it is possible to choose more than one answer):
- heart rate
- respiratory frequency
- digestive sounds
- rectal temperature

Section 3.3. – Pain assessment in sheep

17. When assessing pain in sheep, if you assess pain based on the observation of changes in facial expressions, mark which ones (it is possible to choose more than one answer):
- spasm of the orbital musculature (is there a narrowing of the palpebral fissure present or absent)
- spasm of the masticatory musculature (is there a convex shape in the area of the masseter muscle present or absent)
- abnormal ear position (ventral and caudal rotation)
- abnormal lip and jaw profile (lower jaw profile becomes flat or convex)
- abnormal shape of the nostrils and nose. Philtrum ("V" shape between nostrils)

Section 3.4. – Pain assessment in dogs

18. When assessing pain in dogs, if you assess pain based on behavior, mark which ones (it is possible to choose more than one answer):
- assessment of activity (during rest, whether he is asleep, semi-conscious or awake, restlessness, rolling, self-injury)
- appetite
- mental status (submissiveness, friendly/hostile mood, aggression)
- posture (protection of surgical wound, lateral/sternal position, sitting or standing with head up, standing with relaxed head, moving, abnormal position)
- vocalization (without, during palpation, intermittent, constant)
- attention towards the wound/painful area (does the dog ignore, look, lick, rub, bite the wound/painful area)
- reaction to palpation of the wound/painful area (does not respond, looks around, avoids, growls or protects the area, wants to bite, whines)
- behavior of the dog on a leash (normal, limp, slow or involuntary movement, stiff, refuses to move)

19. If you assess pain in dogs based on physiological parameters, mark which ones (it is possible to choose more than one answer:
- heart rate
- respiratory frequency
- blood pressure
- salivation
- dilated pupils

Section 3.5. – Pain assessment in cats

20. If you assess pain in cats based on behavior, mark which ones (it is possible to choose more than one answer:
- reaction to palpation of the surgical wound (presence or absence of reaction to palpation and abdominal pressure)
- reaction to palpation of the abdomen (presence or absence of reaction to palpation and abdominal pressure)
- does the cat licks and/or bites the wound

- facial expressions (position of ears, orbital musculature, muzzle tension, position of mustache and chin)
- vocalization (does the cat purr when stimulated, meow in the form of interaction with the observer)
- posture (the cat is in a natural position of relaxed muscles; the cat is in a natural position but moves more weakly or refuses; the cat is sitting or in a sternal position with the back hunched and the head down; the cat often changes position)
- comfort (the cat feels comfortable during sleep and also when awake, interaction with stimulation; the cat is calm and slightly sensitive to stimulation; the cat is calm and dissociated from the environment and does not react to stimulation, facing the back of the cage; the cat is uncomfortable, is restless, slightly sensitive to stimulation or dissociated from the environment, facing the back of the cage)
- activity (the cat moves normally, more than normal, calmer than normal or refuses to move)
- mental state (satisfied, uninterested, indifferent, anxious, aggressive)
- appetite

21. If you evaluate pain in cats based on physiological parameters, mark which ones (you can choose more than one answer):
- heart rate
- respiratory frequency
- blood pressure

Section 4 - Veterinarian opinions about owners' ability to assess pain

22. Do you think that owners can reliably assess and interpret pain-induced behavior in their animals? YES NO
23. Do you think that owners can notice pain in his animals better than the veterinarian? YES NO

Section 5 – Pain management

24. Do you think that farm animals are not as sensitive to pain as small animals? YES NO
25. Animals benefit from pain relief? YES NO
26. Is the animal's recovery better if she received analgesic treatment? YES NO
27. Do you think that animals also need analgesia with general anesthesia or sedation? YES NO
28. A certain degree of pain is beneficial, because it reduces the animal's activity after surgery? YES NO
29. Do side effects of analgesics limit their use? YES NO
30. Do owners require analgesics? YES NO
31. Is analgesia affected by the price? YES NO
32. Do you use opioids in your practice? YES NO
33. Mark which opioids you use (you can choose more than one answer): buprenorphine; butorphanol; methadone; pethidine; morphine; tramadol
34. Do you use NSAIDs in your practice? YES NO

35. Mark the NSAIDs you use (you can choose more than one answer): phenylbutazone; flunixin; carprofen; ketoprofen; toclofenamic acid; diprion/hyoscine)
36. Do you use local anesthetics in your practice? YES NO
37. Mark which local anesthetics you use (you can choose more than one answer): lidocaine; bupivacaine, mepivacaine)
38. Do you use for analgesia (you can choose more than one answer): xylazine; detomidine; romifidine; ketamine; second.
39. How often do you use local analgesia: always; often; sometimes; never
40. Do you use epidural techniques? YES NO
41. If you use epidural techniques, mark drugs you use for it (you can choose more than one answer): xylazine; lidocaine; morphine; tramadol, mepivacaine; bupivacaine; detomidine
42. You use epidural techniques for (you can choose more than one answer): perineal surgery; hind limb injuries; foaling and dystocia; rectovaginal dystocia; surgeries in a standing position; perineal lacerations, rectal lacerations/prolapses; uterine prolapse.
43. Do you use other methods for pain relief (multiple answers can be selected): acupuncture; homeopathy; physical therapy; other.
44. Do you use analgesics pre/post-operatively in specific surgical procedures:
- castration: never; sometimes; often; always; I do not perform these procedures
- ovariohysterectomy: never; sometimes; often; always; I do not perform these procedures
45. How long do you use analgesics (single, repeated) in dogs:
- castration: once; repeatedly
- ovariohysterectomy: single, multiple
46. How long do you use analgesics (single, repeated) in cats:
- castration: once; repeatedly
- ovariohysterectomy: single, multiple
47. How long do you use analgesics (single, repeated) in horses:
- castration: once; repeatedly
48. How long do you use analgesics (single, repeated) in cattle:
- castration: once; repeatedly
49. Rate the pain of the procedure:
- castration of a dog: mild, moderate or intense pain
- castration of a cat: mild, moderate or intense pain
- dog ovariohysterectomy: mild, moderate or intense pain
- cat ovariohysterectomy: mild, moderate or intense pain
- horse castration: mild, moderate or intense pain
- castration of livestock: mild, moderate or intense pain
50. What factors influence your choice of analgesics (it is possible to choose more than one answer): price; available information; potential side effects; analgesic potency; market availability.
51. On a scale of 1 to 5, indicate how you consider your knowledge of pain recognition and treatment:
1 – poor
2 – weak
3 – good
4 – very good
5 – excellent

52. What is your main source of information about animal pain (multiple answers are possible): veterinary studies; professional journals; personal experience; regional and national meetings; online webinars.

Your comment or recommendation:

UPITNIK

Dio 1 – Demografski podaci učesnika studije

1. Spol: M Ž
2. Godina diplomiranja na Veterinarskom fakultetu: …. – 2000; 2001 – 2010; 2011- 2020.
3. Najviša akademska titula ili zvanje (godina)?
4. Vrsta prakse: mala praksa, velika praksa, oboje

Dio 2 – Generalna procjena boli

5. Da li smatrate da je teško procijeniti bol kod životinja? DA NE
6. Vaša sposobnost detekcije prisustva boli je: veoma dobra; dobra; umjerena; slaba.
7. Vaša sposobnost kvantifikacije boli: veoma dobra, dobra, umjerena, slaba.
8. Da li koristite tablice/skale za procjenu boli: DA NE
9. Za procjenu prisustva i kvantifikacije boli koristite (moguće odabrati više odgovora): vizualnu analognu tablicu, numeričku tablicu procjene boli, jednostavnu deskriptivnu tablicu, kompozitne tablice za procjenu boli, bihevioralne tablice za procjenu boli, ne koristim tablice za procjenu boli.
10. Da li procjenu boli olakšava poznavanje tipičnog ponašanja životinja? DA NE
11. Smatrate li da na percepciju boli utiče (moguće odabrati više odgovora): vrsta životinje, rasa životinje, individualna senzitivnost, predhodno iskustvo boli i sredine.
12. Da li procjenu prisustva boli i njene kvantifikacije zasnivate na osnovu: promjene ponašanja, promjene fizioloških parametara, kombinacijom navedenih.

Dio 3 – Procjena boli kod konja, krava, ovaca, pasa i mačaka

Dio 3.1. – Procjena boli kod konja

13. Prilikom procjene boli kod konja, ukoliko bol procjenjujete na osnovu ponašanja zaokružite na koja ponašanja obraćate pažnju (moguće odabrati više odgovora)
   - izgled (odbijanje kretanja, nemir, anksioznost, uzbuđenje, depresija)
   - facijalne ekspresije (položaj ušiju, izgled očiju i okolne muskulature, napetost žvakaćih mišića)
   - znojenje
   - udaranje u abdomen
   - kopanje po tlu
   - mahanje repom (ne ubrajati mahanje repom u svrhu tjeranja insekata)
   - položaj (nošenje tjelesne mase, ugodnost)
   - pomjeranje glavom
   - apetit
   - interaktivno ponašanje – obraća pažnju na ljude
   - interaktivno ponašanje – agresija prema ždrijebetu i drugim konjima
   - interaktivno ponašanje – agresija prema ljudima
   - odgovor na palpaciju bolnog područja
   - prisustvo apetita
   - valjanje

- istezanje
- vokalizacija
- struganje zubima
- suzenje
- lateralni položaj
- sopor (duboki san)
- padanje

14. Prilikom procjene boli kod konja, ukoliko bol procjenjujete na osnovu fizioloških parametara, označite kojih (moguće odabrati više odgovora):
    - srčana frekvenca
    - respiratorna frekvenca
    - krvni pritisak
    - digestivni zvukovi
    - rektalna temperatura
    - ishod kliničkog pregleda
    - tip poremećaja

Dio 3.2. – Procjena boli kod krava

15. Prilikom procjene boli kod goveda, ukoliko bol procjenjujete na osnovu ponašanja, označite na koja ponašanja obraćate pažnju (moguće odabrati više odgovora):
    - interaktivno ponašanje – odgovor na taktilne, zvučne i/ili vizuelne stimuluse
    - interaktivno ponašanje – interakcija sa drugim životinjama u blizini
    - lokomocija (način kretanja, odbijanje kretanja ili stajanja)
    - aktivnost (normalno ili smanjeno kretanje, nemir, česte promjene iz ležećeg u stojeći položaj)
    - apetit
    - iznenadno i konstantno mahanje repom
    - lizanje hirurške rane
    - zakrivljenost leđa u toku mirovanja
    - udaranje/stupanje nogama
    - kaudalno istezanje stražnjih ekstremiteta tokom mirovanja
    - glava ispod linije kičmenog stuba
    - ležanje u ventralnom položaju sa potpunom ili parcijalnom ekstenzijom jednog ili oba stražnja ekstremiteta
    - ležanje sa glavom na zemlji
    - ekstenzija vrata i tijela tokom ležanje u ventralnom položaju

16. Prilikom procjene boli kod goveda, ukoliko bol procjenjujete na osnovu fizioloških parametara, označite kojih (moguće odabrati više odgovora):
    - srčana frekvenca
    - respiratorna frekvenca
    - digestivni zvukovi
    - rektalna temperatura

Dio 3.3. – Procjena boli kod ovaca

17. Prilikom procjene boli kod ovaca, ukoliko bol procjenjujete na osnovu observacije promjena facijalnih ekspresija, označite kojih (moguće odabrati više odgovora):

- grč muskulature orbite (da li je prisutno ili odsturno suženje palpebralne fisure)
- grč mastikatorne muskulature (da li je prisutan ili odsutan konveksni oblik u području mišića masetera)
- abnormalan položaj ušiju (rotacija ventralno i kaudalno)
- abnormalan profil usne i vilice (profil donje vilice postaje ravan ili konveksan)
- abnormalan oblik nosnica i nosnog. Filtruma („V“ oblik između nosnica)

Dio 3.4. – Procjena boli kod pasa

18. Prilikom procjene boli kod pasa, ukoliko bol procjenjujete na osnovu ponašanja, označite kojih (moguće odabrati više odgovora):

- procjena aktivnosti (tokom mirovanja da li spava, polusvjestan je ili budan, nemir, valjanje, samoozljeđivanje)
- uzimanje hrane
- mentalni status (submisivnost, prijateljsko/neprijateljsko raspoloženje, agresija)
- položaj u prostoru (protekcija hirurške rane, lateralni/sternalni položaj, sjedi ili stoji sa glavom gore, stoji sa opuštenom glavom, kreće se, abnormalan položaj)
- vokalizacija (bez, prilikom palpacije, intermitentna, konstantna)
- ponašanje prema rani/bolnom području (da li pas ignoriše, gleda, liže, trlja, grize ranu/bolno područje)
- reakcija na palpaciju rane/bolnog područja (ne reaguje, razgleda okolo, izbjegava, reži ili štiti područje, želi ujesti, cvili)
- ponašanje psa na povodcu (normalno, šepa, sporo ili nevoljno kretanje, ukočen, odbija kretanje)

19. Ukoliko bol kod pasa procjenjujete na osnovu fizioloških parametara, označite kojih (moguće odabrati više odgovora:

- srčana frekvenca
- respiratorna frekvenca
- krvni pritisak
- salivacija
- proširene pupile

Dio 3.5. – Procjena boli kod mačaka

20. Ukoliko bol kod mačaka procjenjujete na osnovu ponašanja, označite kojih (moguće odabrati više odgovora:

- reakcija na palpaciju hirurške rane (prisutnost ili odsutnost reakcije na palpaciju i pritisak abdomena)
- reakcija na palpaciju abdomena (prisutnost ili odsutnost reakcije na palpaciju i pritiask abdomena)
- da li mačka liže i/ili grize ranu

- facijalne ekspresije (položaj ušiju, orbitalna muskulatura tenzija njuške, položaj brkova i brade)
- vokalizacija (da li mačka prede pri stimulaciji, mjauče u vidu interakcije sa posmatračem)
- položaj (mačka je u prirodnom položaju relaksiranih mišića; mačka je u prirodnom položaju ali slabije se kreće ili odbija; mačka sjedi ili je u sternalnom položaju sa pogrbljenim leđima i glavom prema dole; mačka često mijenja položaj)
- udobnost (mačka se osjeća udobno tokom sna, a i u budnom stanju, interakcija pri stimulaciji; mačka je mirna i blago osjetljiva na stimulaciju; mačka je mirna i disocirana od okruženja i ne reaguje pri stimulaciji, okrenuta prema stražnjoj strani kaveza; mački je neudobno, nemirna je, blago osjetljiva na stimulaciju ili disocirana od okruženja, okrenuta prema stražnjoj strani kaveza)
- aktivnost (mačka se kreće normalno, više nego normalno, mirnija nego normalno ili odbija da se kreće)
- mentalno stanje (zadovoljna, nezainteresovana, indiferentna, anksiozna, agresivna)
- apetit

21. Ukoliko bol kod mačaka procjenjujete na osnovu fizioloških parametara, označite kojih (moguće odabrati više odgovora):
- srčana frekvenca
- respiratorna frekvenca
- krvni pritisak

Dio 4 – Mišljenje veterinara o spsosobnosti vlasnika da procijeni bol

22. Da li smatrate da vlasnik može pouzdano procijeniti i interpretirati ponašanje uzrokovano boli kod životinja? DA NE
23. Da li smatrate da vlasnik može bolje primijetiti bol kod svojih životinja u odnosu na veterinara? DA NE

Dio 5 – Menadžment boli

24. Da li smatrate da farmske životinje nisu toliko osjetljive na bol kao male životinje? DA NE
25. Životinje imaju korist od olakšavanja boli? DA NE
26. Oporavak životinje je bolji ukoliko je dobila analgetski tretman? DA NE
27. Da li smatrate da je životinjama potrebna i analgezija sa općom anestezijom ili sedacijom? DA NE
28. Određeni stepen boli je koristan, jer smanjuje aktivnost životinja nakon hirurgije? DA NE
29. Da li neželjeni efekti analgetika ograničavaju njihovu upotrebu? DA NE
30. Da li vlasnici zahtijevaju analgetike? DA NE
31. Da li na analgeziju utiče cijena? DA NE
32. Da li koristite opioide u vašoj praksi? DA NE
33. Označite koje opioide koristite (moguće odabrati više odgovora): buprenorfin; butorfanol; metadon; petidin; morfij; tramadol
34. Da li koristite NSAID u vašoj praksi? DA NE
35. Označite NSAID koje koristite (moguće odabrati više odgovora): fenilbutazon; fluniksin; karprofen; ketoprofen; toklofenamična kiselina; diprion/hioscin)

36. Da li koristite lokalne anestetike u vašoj praksi? DA NE
37. Označite koje lokalne anestetike koristite (moguće odabratii više odgovora): lidokain; bupivakain, mepivakain)
38. Da li za analgeziju koristite (moguće odabrati više odgovora): ksilazin; detomidin; romifidin; ketamin; drugo.
39. Koliko često koristite lokalnu analgeziju: uvijek; često; ponekad; nikad
40. Da li koristite epiduralne tehnike? DA NE
41. Epiduralno aplicirate (moguće odabrati više odgovora): ksilazin; lidokain; morfij; tramadol, mepivakain; bupivakain; detomidin
42. Epiduralne tehnike koristite kod (moguće odabrati više odgovora): perinealne hirurgije; povrede stražnjih ekstremiteta; ždrijebljenja i distocije; rektovaginalne distocije; hirurgije u stojećem stavu; perinealne laceracije, rektalne laceracije/prolapsi; uterinog prolapsa.
43. Da li koristite druge metode za ublažavanje boli (moguće je odabrati više odgovora): akupunktura; homeopatija; fizikalna terapija; drugo.
44. Da li koristite analgetike pre/post operativno u specifičnim hirurškim procedurama:
- kastracija: nikad; ponekad; često; uvijek; ne izvodim ove procedure
- sterilizacija: nikad; ponekad; često; uvijek; ne izvodim ove procedure
45. Koliko dugo koristite analgetike (jednokratni, višekratno) kod pasa:
- kastracija: jednokratno; višekratno
- sterilizacija: jednokratno, višekratno
46. Koliko dugo koristite analgetike (jednokratni, višekratno) kod mačaka:
- kastracija: jednokratno; višekratno
- sterilizacija: jednokratno, višekratno
47. Koliko dugo koristite analgetike (jednokratni, višekratno) kod konja:
- kastracija: jednokratno; višekratno
48. Koliko dugo koristite analgetike (jednokratni, višekratno) kod stoke:
- kastracija: jednokratno; višekratno
49. Ocijenite bolnost zahvata:
- kastracija psa: blaga, umjerena ili intenzivna bol
- kastracija mačka: blaga, umjerena ili intenzivna bol
- sterilizacija psa: blaga, umjerena ili intenzivna bol
- sterilizacija mačke: blaga, umjerena ili intenzivna bol
- kastracija konja: blaga, umjerena ili intenzivna bol
- kastracija stoke: blaga, umjerena ili intenzivna bol

50. Koji faktori utiču na izbor analgetika (moguće je odabrati više odgovora): cijena; dostupne informacije; potencijalni neželjeni efekti; analgetska potentnost; dostupnost na tržištu.
51. Na skali od 1 do 5 označite kako smatrate vaše znanje o prepoznavanju i tretmanu boli:
1 – loše
2 – slabo
3 – dobro
4 – veoma dobro
5 – odlično
52. Koji je Vaš glavni izvor informacija o boli životinja (moguće je odabrati više odgovora): veterinarske studije; stručni časopisi; lično iskustvo; regionalni i nacionalni sastanci; online webinari.

Vaš komentar ili preporuka:
